# A Deep-Learning Model for Subject-Independent Human Emotion Recognition Using Electrodermal Activity Sensors

**DOI:** 10.3390/s19071659

**Published:** 2019-04-07

**Authors:** Fadi Al Machot, Ali Elmachot, Mouhannad Ali, Elyan Al Machot, Kyandoghere Kyamakya

**Affiliations:** 1 Research Center Borstel—Leibniz Lung Center, 23845 Borstel, Germany; 2 Faculty of Mechanical and Electrical Engineering, University of Damascus, Damascus, Syria; ali.elmachot@gmail.com; 3 Institute for Smart Systems Technologies, Alpen-Adira University, 9020 Klagenfurt, Austria; mouhannad.ali@aau.at (M.A.); kyandoghere.kyamakya@aau.at (K.K.); 4 Carl Gustav Carus Faculty of Medicine, Dresden University of Technology, 01069 Dresden, Germany; Elyan.Al-Machot@uniklinikum-dresden.de

**Keywords:** subject-dependent emotion recognition, subject-independent emotion recognition, electrodermal activity (EDA), deep learning, convolutional neural networks

## Abstract

One of the main objectives of Active and Assisted Living (AAL) environments is to ensure that elderly and/or disabled people perform/live well in their immediate environments; this can be monitored by among others the recognition of emotions based on non-highly intrusive sensors such as Electrodermal Activity (EDA) sensors. However, designing a learning system or building a machine-learning model to recognize human emotions while training the system on a specific group of persons and testing the system on a totally a new group of persons is still a serious challenge in the field, as it is possible that the second testing group of persons may have different emotion patterns. Accordingly, the purpose of this paper is to contribute to the field of human emotion recognition by proposing a Convolutional Neural Network (CNN) architecture which ensures promising robustness-related results for both subject-dependent and subject-independent human emotion recognition. The CNN model has been trained using a grid search technique which is a model hyperparameter optimization technique to fine-tune the parameters of the proposed CNN architecture. The overall concept’s performance is validated and stress-tested by using MAHNOB and DEAP datasets. The results demonstrate a promising robustness improvement regarding various evaluation metrics. We could increase the accuracy for subject-independent classification to 78% and 82% for MAHNOB and DEAP respectively and to 81% and 85% subject-dependent classification for MAHNOB and DEAP respectively (4 classes/labels). The work shows clearly that while using solely the non-intrusive EDA sensors a robust classification of human emotion is possible even without involving additional/other physiological signals.

## 1. Introduction

Emotion recognition plays an important role in various areas of life, especially in the field of Active and Assisted Living (AAL) [1] and Driver Assistance Systems (DAS) [2]. Recognizing emotions automatically is one of technical enablers of AAL, as it is considered to be a significant help for monitoring and observing the mental state of either old people or disabled persons.

Furthermore, it can be observed that according to the most recent related publications, the classification performance of emotion recognition approaches has been significantly improving and the opportunities for automatic emotion recognition systems are also getting higher.

Emotions can be recognized in various ways. The most well-known models for emotion recognition are the “discrete emotion model” proposed by Ekman [3] and the “emotion dimensional model” proposed by Lang [4]. The discrete emotion model categorizes emotions into six basic emotion states: surprise, anger, disgust, happiness sadness and fear [3]. These emotions are universal, biologically experienced by all humans and widely accepted as such in the research community. In contrast to the discrete emotional model, the dimensional model assumes that the emotions are a combination of several psychological dimensions. The most well-known dimensional model is the “valance-arousal dimensional model”. The valance represents a form of pleasure level and ranges from negative to positive. However, the arousal indicates the physiological and/or psychological level of being awake and ranges from low to high [5].

Overall, researchers in the field have used two major approaches to recognize emotions. The first one consists of features engineering-based approaches [6] and the second one involves Deep Learning (DL) [7]. In the features engineering approach, human emotion recognition involves several steps ranging from collecting raw sensor data up to the final conclusion about the current emotional status. The steps thereby involved are the following ones [8]: (1) preprocessing of the raw data from sensor streams for handling incompleteness, eliminating noise and redundancy, and performing data aggregation and normalization; (2) feature extraction which means extracting the main characteristics of/from the raw signals (e.g., temporal and spatial information); (3) dimensionality reduction to decrease the number of features to increase their quality and reduce the computational effort needed for the classification task; and (4) classification based on machine-learning and reasoning techniques to recognize the effective emotion class.

On the other hand, DL does not require necessarily the feature engineering/extraction step, due to the fact that DL models do extract features internally and/or implicitly (within the training phase) [9]. Therefore, they have shown promising results while involving a combination of different physiological signals for human emotion recognition [10,11].

Additionally, DL showed promising results in other research fields for different applications, e.g., identification of gas mixture [12], classification of tea specimens [13] and cardiac arrhythmia detection [14,15].

Generally, subject-independent emotion recognition is a challenging field due to the facts that (a) physiological expressions of emotion depend on age, gender, culture and other social factors [16], and (b) it also depends on the environment in which a subject lives, (c) the subject-independent nature of human emotion recognition which means that the system has been trained on a group of subjects and tested on another different group, and (d) the lab-setting independent nature of emotion recognition is related to the fact that the classifier can/will be trained locally once using sensors of a given lab-setting and after that tested considering different datasets that are collected based on different lab settings. The motivation for developing a generalized model is that collecting training data each time for each subject is not a realistic task and is far from the practical reality.

Based on the previous facts, a concept to improve the performance of the subject-dependent and subject-independent human emotion recognition systems is required; in this paper we use solely EDA (electrodermal activity) biosignals based on a deep-learning model using convolutional neural networks (CNNs) that extracts the required features internally and performs well when this model is applied on new subjects. Although researchers have used CNN to classify human emotions using EDA, they did not propose the architecture that did perform better than the proposed model in this paper.

The contribution of this paper does significantly increase the performance of human emotion recognition approaches using only EDA sensors compared to the state-of-the-art approaches involving the same EDA signals. Furthermore, the results obtained suggest/underscore a novel fact and interesting situation that other (mostly “highly intrusive”) physiological sensors might be replaced by the “only slightly intrusive” EDA-based sensors in this research field. The structure of the paper is as follows: Section 2 presents an overview of the state-of-the-art approaches. Section 3 introduces the datasets. Section 4 portrays the overall architecture of the proposed classification model. Section 5 and Section 6 present the overall results and the related discussions respectively. The paper ends with a conclusion in Section 7.

## 2. Related Works

Regarding human emotion recognition based on EDA sensors which can be embedded in smart wearable devices, few works have been published so far. However, in [17], they proposed a system to recognize the driver’s emotional state after transforming the EDA signals using a short-time Fourier transform. They considered three classes: neutral-stress, neutral-anger, and stress-anger.

Furthermore, in [18], they applied a convex optimization-based electrodermal activity (cvxEDA) framework and clustering algorithms to automatically classify the arousal and valence levels induced by affective sound stimuli.

In the literature, it has been proven that the stimuli nature plays an important role to increase the EDA response which helps to make the emotion recognition process less complex [19]. Furthermore, other works showed promising results when EDA responses are modulated by musical emotional [20,21]. Consequently, this result encouraged researchers to work on classifying arousal and valence levels induced by auditory stimuli.

In [22], authors used the AVEC 2016 dataset [23,24], they proposed a deep-learning model that consists of a CNN followed by a recurrent neural network and then fully connected layers. They showed that an end-to-end deep-learning approach directly depending on raw signals can replace feature engineering for emotion recognition purposes.

Moreover, the use of different physiological signals has been previously involved [25,26]. However, mounting different types of sensors on the human body is not preferred and nor well-accepted. In [26], authors fused different types of sensors, ECG (Electrocardiogram), EDA and ST (Skin Temperature) through a hybrid neural model which combines cellular neural networks and echo state neural networks to recognize four classes of valence and arousal, mainly, high valence high arousal, high valence low arousal, low valence high arousal, and low valence, low arousal. In [25], authors combined facial electromyograms, electrocardiogram, respiration, and EDA dataset which were collected during racing conditions. The emotional classes identified are high stress, low stress, disappointment, and euphoria. Support vector machines (SVMs) and adaptive neuro-fuzzy inference system (ANFIS) have been used for the classification.

In [27], the researchers reported results using only EDA to recognize four different states, joy, anger, sadness, pleasure using 193 features and a music and based on genetic algorithm and the K-neighbor methods.

Table 1 shows a summary of the state-of-the-art for human emotion recognition using physiological signals. More details regarding state-of-the-art experiments and obtained results can be found in Section 6.

The major limitations in the state-of-the-art can be summarized in three major points. First, the limitation regarding proposing generalized models to recognize human emotions based on EDA signals (i.e., published works do not comprehensively consider the lab-setting independence property of emotion classifiers for EDA signals). Second, the limitation concerning subject-independent human emotion recognition (i.e., published works do not comprehensively address the subject-independence property of emotion classifiers for EDA signals). Third, most published related works do focus mostly on classifying only 2 (active/passive) emotional states.

In this work, we focus on the second and the third limitation, due to the fact that classifying human emotion with respect to different lab settings is a research question which may need to adjust the raw data in a feature engineering level which is not the focus of this work where CNN does extract the desired features internally as it is a deep-learning model.

## 3. Datasets

This study uses public benchmark datasets (MAHNOB and DEAP) of physiological signals to test our proposal for a robust emotion recognition system. However, for both solely the EDA related data will be used in the experiments for this paper.

### 3.1. MAHNOB

The dataset used is called MAHNOB and was collected by Soleymani Mohammad et al. [31]. The data is related to different physiological signals.

**Figure 1:**
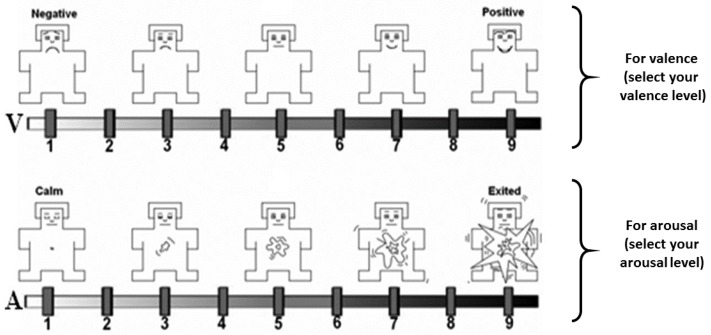
Self-assessment manikins scales for valence (above) and arousal (below) [32].

The data was collected from 30 young healthy adults who participated in the study. 17 of the participants were female and 13 of them were males. Their age varied between 19 to 40. The participants were shown 20 emotional video clips which were evaluated in terms of both valence and arousal by using the Self-Assessment Manikins (SAM) questionnaire [32]. SAM is a prominent tool that visualizes the degree of valence and arousal by manikins. The participants distinguished a scale from 1 to 9, see Figure 1.

In the experiments for MAHNOB, electroencephalogram (EEG), blood volume pressure (BVP), respiration pattern, skin temperature, electromyogram (EMG), electrooculogram (EOG), electrocardiogram (ECG), and EDA of 30 participants were collected.

### 3.2. DEAP

DEAP [33] is a multimodal dataset used to analyze human emotional states.

The stimuli used in the experiments were chosen in different steps. First, they selected 120 initial stimuli that were selected both semi-automatically and manually. Second, a one-minute highlight part was specified for each stimulus. Third, through a web-based subjective assessment experiment, 40 final stimuli were chosen.

During the physiological experiment, 32 participants evaluated 40 videos via a web interface used for subjective emotion assessment in terms of the levels of arousal, valence, like/dislike, dominance, and familiarity. The age of participants varied between 19 to 37. Concerning the classes/labels for DEAP, we considered the same classes as same as in Section 4.1.

In the experiment, electroencephalogram (EEG), BVP, respiration pattern, ST, electromyogram (EMG), electrooculogram (EOG), electrocardiogram (ECG), and EDA of 32 participants were collected.

## 4. Classification Using a Convolution Neural Network—CNN

In this section, we present, the labelling of EDA signals, the design details of the proposed CNN for emotion classification and then, the evaluation metrics and evaluation.

### 4.1. Preprocessing and Labelling

First, raw data of EDA were scaled such that the distribution is centered around 0, with a standard deviation of 1. Additionally, after data normalization, two states [34] valence and arousal are addressed for emotion classification. In this regard, the scales (1–9) were mapped into 2 levels for each valence and arousal state according to the SAM ratings.

The valence scale of 1–5 was mapped to “negative” and 6–9 to “positive”, respectively. The arousal scale of 1–5 was mapped to “passive” and 6–9 to “active”, respectively.

- **High Valence/High Arousal (HVHA)**. This class includes positive emotions such as happy and excited.
- **High Valence/Low Arousal (HVLA)**. This class includes emotions such as relaxed, calm and pleased.
- **Low Valence/High Arousal (LVHA)**. This class includes emotions such as anger, fear and distressed.
- **Low Valence/Low Arousal (LVLA)**. This class includes negative emotions such as sad and depressed.

### 4.2. Classifiers

To perform the emotions classification task, we propose a deep-learning approach. A CNN is a kind of feedforward network structure that consists of multiple layers of convolutional filters followed by subsampling filters and ends with a fully connected classification layer. The classical LeNet-5CNN first proposed by LeCun et al. in [35] is the basic model for various CNN applications for object detection, localization, and prediction.

First, the EDA signals are converted into matrices whereby the goal is to make the application of CNN model possible (see Section 5).

**Figure 2:**
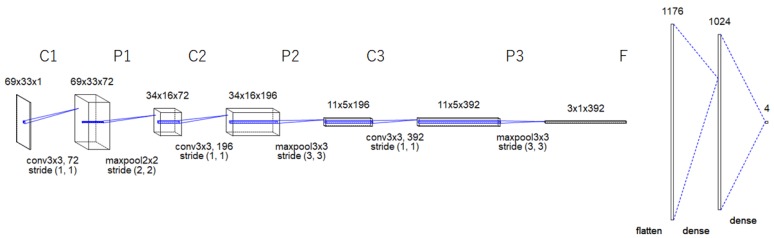
The proposed CNN model.

As illustrated in Figure 2, the proposed CNN architecture has three convolutional layers (C1, C2, and C3), three subsampling layers in between (i.e., P1, P2, and P3), and an output layer F.

The convolutional layers generate feature maps using 72 (3 × 3) filters followed by a Scaled Exponential Linear Units (SELU) as an activation function, 196 (3 × 3) filters followed by a Rectified linear unit (ReLU) as an activation function and 392 (3 × 3) filters followed by a ReLU as an activation function.

Additionally, in the subsampling layers, the generated feature maps are spatially down-sampled. In our proposed model, the feature maps in layers C1, C2 and C3 are sub-sampled to a corresponding feature map of size 2 × 2, 3 × 3 and 3 × 3 in the subsequent layers P1, P2, and P3 respectively.

The output layer F is a fully connected neural model that performs the classification process, it consists of three layers. The first layer has 1176 nodes, each activated by a ReLU activation function. The second layer has 1024 nodes, each activated by a SELU activation function. The final layer is the SoftMax output layer C1.

The result of the mentioned layers is a 2D representation of extracted features from input feature map(s) based on the input EDA signals.

Since the dropout is a regularization technique to avoid over-fitting in neural networks based on preventing complex co-adaptations on training data [36], therefore, our dropout for each layer was 0.25 which is related to a fraction of the input units to drop. Table 2 shows parameters used for all the layers of the proposed CNN model.

A grid search technique has been used to fine-tune the CNN model hyperparameters and to find out the optimal number of filters and layers needed to perform the emotion classification task. We have used the GridSearchCV class in Scikit-learn [37]. We have provided a dictionary of hyperparameters that should be checked during the performance evaluation. By default, the grid search uses one thread, but it can be configured to use all available cores to increase the calculation time. Then, the Scikit-learn class has been combined with Keras to find out what are the best hyperparameters values. Additionally, cross a validation is used to evaluate each individual model and the default of 10-fold cross-validation has been used.

All provided results have been obtained while using the following computer platform: Intel Corei7-7820HK processor Quad-Core 2.90 GHz, 16 GB DDR4 SDRAM, NVIDIA GeForce GTX 1080 with 8 GB dedicated storage.

Additionally, we examine several classifiers to compare the performance of the existing models with that of the here proposed one. In particular, Support Vector Machine (SVM) [38], K-Nearest Neighbor (KNN) [39], Naive Bayes [40] and Random Forest [41] are considered for benchmarking.

**Figure 3:**
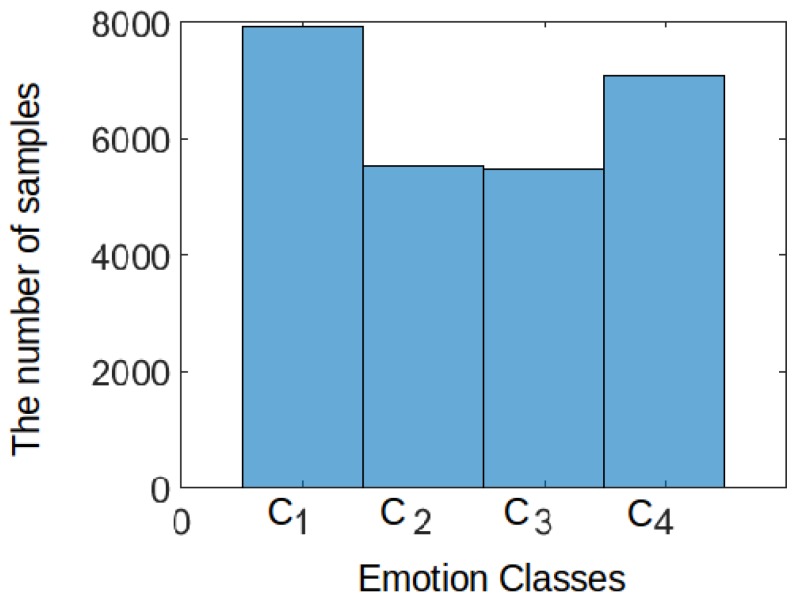
Overall emotion distribution for one Subject, where C1: High Valence/High Arousal (HVHA), C2: High Valence/Low Arousal (HVLA), C3: Low Valence/Low Arousal (LVLA) and C4: Low Valence/High Arousal (LVHA) based on a subject’s data in MAHNOB.

**Figure 4:**
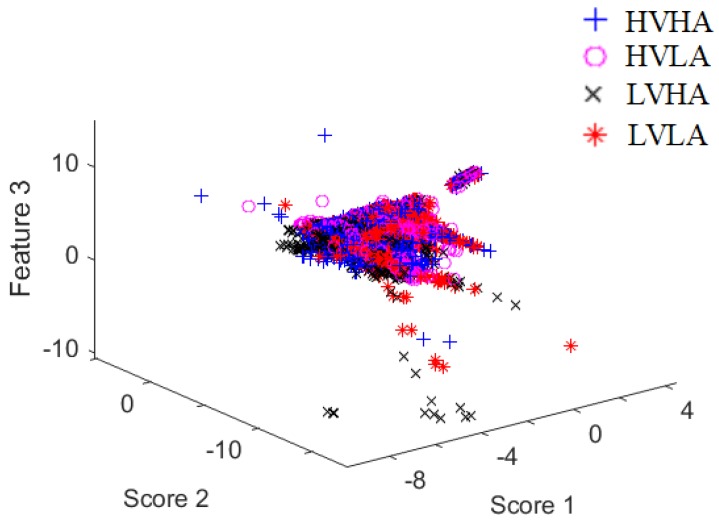
Scatter plot of the first three Fisher scores based on a subject’s data in MAHNOB.

Based on Figure 3 and Figure 4, selecting the previous classifiers has different advantages for comparison purposes. For example, the objective of random forests is that they consider a set of high-variance, low-bias decision trees and convert them into a model that has both low variance and low bias. On the other hand, KNNs is an algorithm which stores all the available cases and classifies new cases based on a similarity measure (e.g., distance functions). Therefore, KNN has been applied in statistical estimation and pattern recognition from the beginning of the 1970s on as a non-parametric technique [39]. Support Vector Machines are well-known in handling non-linearly separable data based on their non-linear kernel, e.g., the SVM with a polynomial kernel (SVM (poly)), and the SVM with a radial basis kernel (SVM (rbf)). Therefore, we classify the EDA data using three types of SVMs, namely the following ones: SVM (linear) (i.e., standard linear SVM), SVM (poly) and SVM (rbf). Finally, we used a simple probabilistic model which is the Naive Bayes. The purpose of using such a probabilistic model is to show how it behaves on EDA data. Table 3 shows the values of parameters of proposed CNN and other classifiers.

### 4.3. Evaluation Metrics and Validation Concept

To evaluate the overall performance of the classifiers, we consider several performance metrics. In particular, we use precision, recall, f-measure, and accuracy, as in [42].

The Equations (1)–(4) show mathematical expressions of the metrics precision, recall, accuracy, and f-measure respectively, where TP, TN, FP, and FN refer respectively to “True Positives”, “True Negatives”, “False Positives” and “False Negatives” respectively.

(1) $$Precision=\frac{\mathrm{TP}}{\mathrm{TP}\;+\;\mathrm{FP}}$$

(2) $$Recall=\frac{\mathrm{TP}}{\mathrm{TP}\;+\;\mathrm{FN}}$$

(3) $$Accuracy=\frac{\mathrm{TP}\;+\;\mathrm{TN}}{\mathrm{TP}\;+\;\mathrm{TN}\;+\;\mathrm{FN}\;+\;\mathrm{FP}}$$

(4) $$F1=\frac{2\cdot precision\cdot recall}{precision+recall}$$

Regarding the evaluation scenarios, we consider two cases. The subject-dependent and subject-independent cases. Subject-dependent means training and testing have been performed on the same subject. Subject-independent means the training has been performed on a group of subjects and testing has been performed on a totally new group of subjects.

## 5. Results

To have a deeper understanding of the performance of the proposed CNN model, MAHNOB, and DEAP datasets were used for testing the overall classification performance.

Moreover, the data distribution should be taken into consideration to choose a suitable classifier for comparison purposes. In this regard, a Fisher mapping [43] was used to define the three major scores in the samples that are investigated. Based on the output of Figure 3 and Figure 4, it is concluded that the data is highly overlapped, and there is a kind of class imbalance problem.

In this assessment, 10 subjects were selected from the MAHNOB and DEAP datasets. Each dataset for each subject consists of four classes (see Section 3.1 and Section 3.2). The average training time for each subject was approximately 21 min.

The length of the considered EDA signals is 2574 that are converted to matrices of size (39 × 66). All results are presented for ten-fold cross-validation.

Table 4 and Table 5 present the average values for the precision, the recall, and the f-measure using DEAP and MAHNOB datasets respectively. The tables show the performance metrics values when training and testing are performed on the same subject. The tables show the average value of precision, recall, and f-measure with respect to each subject. The performance metrics values for each subject have been summed and divided by the total number of subjects. The major target of this experiment is to check out the overall performance for subject-dependent EDA-based emotion classification.

Table 6 and Table 7 present the precision the recall, and the f-measure using DEAP and MAHNOB datasets respectively. The results are obtained when training and testing are performed on different subjects. The major target of this experiment is to check out the overall performance for subject-independent EDA-based emotion classification.

In all tables, the proposed CNN model shows the highest performance compared to K-NN and random forest which are hereby the best next two classifiers. When K-NN and random forest classifiers perform well, it indicates that the dataset is not easily separable, and the nonlinearity is high. This can be observed in Figure 4. Accordingly, the decision planes generated using other classifiers (see Table 4, Table 5, Table 6 and Table 7) do not categorize some points in space to an inappropriate region as good as K-NN and random forest classifiers.

The performance metrics and the implementation are written in Python using Numpy (http://www.numpy.org/), Scikit-learn (https://scikit-learn.org/) and Keras (https://keras.io/). All performance metrics are calculated for each class and weighted taking the class imbalance into account. Accordingly, the evaluation metrics for each label have been calculated and their average has been weighted by the support measurement which is the number of true instances for each label.

Table 8 and Table 9 show the confusion matrix for both MAHNOB and DEAP (the average performance results for training and testing on same subjects) and the confusion matrix for both MAHNOB and DEAP (the average performance results for training and testing on different subjects), respectively.

## 6. Discussion

Aiming at highlighting the contribution of this work, other works should be considered and analyzed. However, it is not easy to make such a comparison due to the fact that (a) other works may combine other types of physiological signals and they do not use only EDA, and (b) the reaction and the response of EDA does highly depend on the stimuli type, which showed better results when the stimuli is an acoustic one [18].

To our knowledge, this study shows for the first time that developing a subject-independent human emotion recognition using only EDA signals with a promising recognition rate is possible. It is also worthwhile noting that we were able to,

- increase the f-measure for subject-independent classification to 78% and 81% for MAHNOB and DEAP respectively (4 classes/labels).
- increase the f-measure for subject-dependent classification have been increased to 83% and 85% for MAHNOB and DEAP respectively (4 classes/labels).

In the state-of-the-art, researchers in [22] tested a deep-learning model which consists of RNN and CNN which showed a Concordance Correlation Coefficient (CCC) [44] of 0.10 on the arousal dimension and 0.33 on the valence dimension based on EDA only. They used AVEC 2016 dataset [23,24].

In addition, in [27], they reported an emotion recognition analysis using only the EDA signal for subject-dependent with an accuracy of 56.5% for the arousal dimension and 50.5% for the valence dimension based on four songs stimuli. In [18], authors suggested a system which can achieve a recognition accuracy of 77.33% on the arousal dimension, and 84% on the valence dimension based on three emotional states induced by affective sounds taken from IADS collection [45].

Furthermore, it should be mentioned that the binary classification (passive/active cases) of EDA signals showed high results as in [28] with an accuracy of 95% using SVM and an accuracy of 80% using CNNs in [29].

However, getting such a high performance for two classes is expected where other studies showed clearly that EDA signals for active and passive states form clear patterns compared to the 4 classes of arousal and valence for emotion recognition [46]. Table 10 shows a summary of the state-of-the-art for EDA-based emotion detection regarding, experiment, number of classes, used classifiers, and the reported accuracy.

Additionally, analyzing the results of the state-of-art, clearly, feature engineering for subject-independent and subject-dependent human emotion detection based on EDA does not lead to high performance. In particular, when the number of classes is higher than two. This is because extracting the sympathetic response patterns which are part of each emotion is difficult. Furthermore, when trying to overcome this fact by analyzing more basic features such as level, response amplitude, rate, rise time, and recovery time, they discard flexible elicited behavior which might improve emotion recognition. Therefore, it has been proven in this work that DL can overcome this drawback quite well.

Regarding the point of testing the proposed model using different datasets from different labs, it is because human emotions do not form similar patterns. Consequently, the research community should develop generalized models to recognize human emotions, where subjects, elicitation materials, and physiological sensors brands are different from the ones involved in the initial training. Dealing with such research question has an important impact for human support in the frame of smart environments in different applications.

Concerning, human emotion recognition with respect to different lab–settings, in [30], authors showed that adjusting and manipulating the feature space to bring both datasets to a homogeneous feature space as a pre–processing step may increase the overall performance even when datasets come from different labs.

Moreover, in [47], they checked the ability of 504 school children aged between 8 and 11 years old to recognize the emotions of facial expressions based on pictures. The overall performance was approximately 86% to recognize anger, fear, sadness, happiness, disgust, and neutral facial expressions. It is impressive to see that the proposed automated EDA-based emotion recognition system is close to the performance of human capability to interpret the facial expressions.

## 7. Conclusions

This study can be considered to be a basic contribution in terms of overcoming the generalization problem for human emotion recognition. The aim was to show the feasibility and the possibility of building such generalized models for relevant application contexts. Furthermore, this study examined the less intrusive sensors based on statistical analyses in real-life datasets and reviewed various state-of-the-art approaches to human emotion recognition in smart home environments.

Additionally, emotion recognition is a cornerstone of advanced intelligent systems for monitoring a subject’s comfort. Thus, information on a subject’s emotion and stress level is a key component for the future of smart AAL environments.

In our future work, we will focus on human emotion recognition using EDA with respect to different lab–settings, which means, we will try to build a generalized approach which should be trained using lab–settings X and tested using lab–settings Y. Additionally, we plan to combine Stacked Sparse Auto Encoders with CNN. Moreover, CNN essentially learns local (spatial) features. On the other side, RNN does in essence rather learn temporal features. Consequently, combining both neural network concepts will result in a neuro-processor which can learn both contextual dependencies (i.e., spatial and temporal) from inputted local features. As a result, such a combination does potentially improve the overall performance.

## Figures and Tables

**Table 1:** Summary of the stare-of-the-art works for human emotion recognition using physiological signals.

Paper	Classifier	Features	Signals
[25]	SVM	Statistical Features	Facial electromyograms, electrocardiogram, respiration, and electrodermal activity
[27]	Genetic algorithm and K-NN	Statistical features	EDA
[25]	Neuro-fuzzy inference	Statistical Features	Facial electromyograms, electrocardiogram, respiration, and electrodermal activity
[18]	K-NN	Statistical features	EDA
[28]	SVM	Wrapper feature selection (WFS)	EDA
[29]	CNN	Raw data	Patient’s movements XYZ + EDA
[22]	Deep learning (CNN+RNN)	Raw data	AVEC 2016
[26]	ESN-CNN	Statistical features	ECG (Electrocardiogram), EDA (Electrodermal activity) and ST (Skin Temperature)
[30]	Dynamic calibration + K-NN	Statistical features	EDA

SVM: Support Vector Machine, K-NN: K-Nearest Neighbor, CNN: Convolutional Neural Network, RNN: Recurrent Neural Network, ESN-CNN: Echo State Network - Cellular Neural Network.

**Table 2:** Parameters used for all the layers of the proposed CNN model.

Layer	Kernel, Units	Other Layers Parameters
C1	(3 × 3), 2	Activation = Selu, Strides = 1
P1	(2×2)	Strides = 2
C2	(3×3), 196	Activation = Selu, Strides = 1
P2	(3×3)	Strides=3
C3	(3×3)3, 92	Activation = Selu, Strides = 1
P3	(3×3)	Strides = 3

C is the convolution layer, P is the max-pooling layer and SELU is the Scaled Exponential Linear Unit activation function.

**Table 3:** Values of parameters of proposed CNN and other classifiers.

Model	Parameters
SVM (poly)	Degree of the polynomial kernel function = 3, $\gamma =\frac{1}{number\;of\;features}$
SVM (rbf)	$\gamma =\frac{1}{number\;of\;features}$
Random Forest	Number of estimators estimators = 10 trees, criterion = Gini impurity, The minimum number of samples required to split an internal node = 2
Naive Bayes	Prior = probabilities of the classes
KNN	Distance metric = ’minkowski’, Power parameter for the Minkowski metric = 2, Number of neighbors = 3
Proposed (CNN)	Loss = categorical_crossentropy, optimizer = Adam, batch_size = 50, epochs = 1000

**Table 4:** Performance metrics for DEAP (the average performance results for training and testing on same subject).

Model	Accuracy	Precision	Recall	F-Measure
SVM (Linear)	0.46	0.41	0.46	0.42
SVM (poly)	0.41	0.53	0.43	0.33
SVM (rbf)	0.59	0.60	0.60	0.58
Random Forest	0.74	0.76	0.75	0.75
Naive Bayes	0.44	0.48	0.44	0.42
K-NN	0.80	0.80	0.80	0.80
**Proposed CNN**	**0.85**	**0.85**	**0.85**	**0.85**

**Table 5:** Performance metrics for MAHNOB (the average performance results for training and testing on same subject).

Model	Accuracy	Precision	Recall	F-Measure
SVM (Linear)	0.49	0.48	0.50	0.43
SVM (poly)	0.47	0.49	0.48	0.36
SVM (rbf)	0.55	0.53	0.56	0.51
Random Forest	0.68	0.70	0.70	0.70
Naive Bayes	0.37	0.43	0.39	0.35
K-NN	0.74	0.76	0.75	0.75
**Proposed CNN**	**0.81**	**0.81**	**0.81**	**0.81**

**Table 6:** Performance metrics for MAHNOB (the average performance results for training and testing on different subjects).

Model	Accuracy	Precision	Recall	F-Measure
SVM (Linear)	0.34	0.47	0.34	0.37
SVM (poly)	0.36	0.70	0.37	0.42
SVM (rbf)	0.41	0.53	0.42	0.45
Random Forest	0.64	0.65	0.65	0.65
Naive Bayes	0.27	0.43	0.27	0.33
K-NN	0.72	0.73	0.73	0.72
**Proposed CNN**	**0.78**	**0.78**	**0.78**	**0.78**

**Table 7:** Performance metrics for DEAP (the average performance results for training and testing on different subjects).

Model	Accuracy	Precision	Recall	F-Measure
SVM (Linear)	0.40	0.41	0.40	0.31
SVM (poly)	0.39	0.41	0.39	0.28
SVM (rbf)	0.44	0.50	0.44	0.40
Random Forest	0.69	0.70	0.69	0.69
Naive Bayes	0.36	0.31	0.36	0.28
K-NN	0.75	0.76	0.75	0.76
**Proposed CNN**	**0.82**	**0.83**	**0.82**	**0.83**

**Table 8:** Confusion matrix for both MAHNOB and DEAP (the average performance results for training and testing on same subjects).

Class	C1	C2	C3	C4
C1	0.861	0.057	0.071	0.046
C2	0.062	0.808	0.059	0.034
C3	0.039	0.050	0.878	0.017
C4	0.045	0.063	0.042	0.866

C1: High Valence/High Arousal (HVHA), C2: High Valence/Low Arousal (HVLA), C3: Low Valence/Low Arousal (LVLA) and C4: Low Valence/High Arousal (LVHA).

**Table 9:** Confusion matrix for both MAHNOB and DEAP (the average performance results for training and testing on different subjects).

Class	C1	C2	C3	C4
C1	0.762	0.177	0	0.146
C2	0.049	0.685	0	0.077
C3	0.004	0	0.705	0.017
C4	0.108	0.126	0.058	0.857

C1: High Valence/High Arousal (HVHA), C2: High Valence/Low Arousal (HVLA), C3: Low Valence/Low Arousal (LVLA) and C4: Low Valence/High Arousal (LVHA).

**Table 10:** A summary of the state-of-the-art results using only EDA.

Paper	Experiment	Number of Classes	Classifier Used	Arousal	Valence	Accuracy (Both)
[27]	Subject-dependent	4	Genetic algorithm and K-NN	0.56	0.50	–
[18]	Subject-independent	3	K-NN	0.77	0.84	–
[28]	Subject-independent	2	SVM	–	–	0.95
[29]	Subject-dependent	2	CNN	–	–	0.80
[22]	Subject-independent	2	CNN	0.10	0.33	–
Proposed CNN	Subject-independent (DEAP)	4	CNN	–	–	**0.82**
Proposed CNN	Subject-independent (MAHNOB)	4	CNN	–	–	**0.78**
Proposed CNN	Subject-dependent (DEAP)	4	CNN	–	–	**0.85**
Proposed CNN	Subject-dependent (MAHNOB)	4	CNN	–	–	**0.81**

SVM: Support Vector Machine, K-NN: K-Nearest Neighbor, CNN: Convolutional Neural Network.
